# Prognostic Value of Beta-Tubulin-3 and c-Myc in Muscle Invasive Urothelial Carcinoma of the Bladder

**DOI:** 10.1371/journal.pone.0127908

**Published:** 2015-06-05

**Authors:** Francesco Massari, Emilio Bria, Chiara Ciccarese, Enrico Munari, Alessandra Modena, Valentina Zambonin, Isabella Sperduti, Walter Artibani, Liang Cheng, Guido Martignoni, Giampaolo Tortora, Matteo Brunelli

**Affiliations:** 1 Medical Oncology d.U., Azienda Ospedaliera Universitaria Integrata (AOUI) and University of Verona, Verona, Italy; 2 Department of Pathology and Diagnostic, Azienda Ospedaliera Universitaria Integrata (AOUI) and University of Verona, Verona, Italy; 3 Biostatistics, Regina Elena National Cancer Institute, Rome, Italy; 4 Urologic Clinic, Department of Oncological and Surgical Sciences, Azienda Ospedaliera Universitaria Integrata (AOUI) and University of Verona, Verona, Italy; 5 Department of Pathology and Laboratory Medicine, Indiana University School of Medicine, Indianapolis, Indiana, United States of America; Istituto dei tumori Fondazione Pascale, ITALY

## Abstract

**Background:**

To date, putative prognostic biomarkers have shown limited utility from the clinical perspective for bladder urothelial carcinoma. Herein, the expression of beta-tubulin-3 and c-Myc was evaluated to determine their prognostic potential.

**Methods:**

In formalin fixed-paraffin embedded blocks, immunohistochemical expression of c-Myc and beta-tubulin-3 was evaluated. H score ranging from 0 to 300 was obtained by multiplying the percentage of positive cells by intensity (0–3); c-Myc and beta-tubulin-3 expression was defined: 0: negative, 1: weakly positive, 2: strongly positive.

**Results:**

beta-tubulin-3 and c-Myc immunoexpression was available for 46 cases. At the univariate analysis, node-involvement, beta-tubulin-3 and c-Myc overexpression discriminate shorter DFS (HR 2.19, p = 0.043; HR 3.10, p = 0.24 and HR 3.05, p = 0.011, respectively); 2-yrs DFS log-rank analysis according to low versus high level of immunoexpression were statistically significant; beta-tubulin-3, 53% low vs 12.7% high (p = value 0.02) and c-Myc 28 low vs 8 high (p-value 0.007). Patients displaying negative beta-tubulin-3/c-Myc had statistically significant better 2-yrs DFS than those with mixed expression or double positivity (54.5% versus 18.7% versus 0%, log-rank p = 0.006).

**Conclusions:**

c-Myc and beta-tubulin-3 show improvement for prognostic risk stratification in patients with muscle invasive bladder urothelial carcinoma. These molecular pathways may also be candidate to improve predictiveness to targeted therapies.

## Introduction

Urothelial carcinoma is the 5th most common cancer in industrialized countries, accounting for approximately 5% of all cancers and represents the second most common genitourinary malignancy [1,2]. At the time of diagnosis, approximately 70% of patients present with superficial disease and the remaining 30% with carcinoma infiltrating the muscolaris tonaca. Of this group about one third of the patients present with metastatic disease not visible at the time of treatment of the primary tumor. Moreover, approximately 50% of patients undergoing radical cystectomy for invasive bladder cancer develops a local recurrence or distant, while roughly 10%-15% is presented with metastatic disease at diagnosis.

Currently there are no valid tissue-based biomarkers that serve as useful molecular prognostic factors to guide the clinical management of the patient with carcinoma of the bladder [3,4]. The most crucial treatment decisions are based on clinical risk factors and histopathological criteria. Overall, the management of these patients may derive great benefit from the identification and use of markers reproducible and objective, able to predict the risk of relapse and progression, thus allowing individualization of treatment. In addition, to reduce the recurrence rate, limit the progression of superficial disease and increase survival and quality of life of patients with invasive or metastatic disease, would prove useful new therapeutic approaches to be used alone or in combination with conventional treatment.

In different districts, overexpression of beta-tubulin-3 in cancer cells has been associated with resistance to docetaxel-based chemotherapy in patients with advanced cancer [5,6] and recent inhibitors targeting the c-Myc pathways has been proposed.

Aim of our study was Digita il testo o l'indirizzo di un sito web oppure to evaluate the correlation between these pathways (c-Myc and beta-tubulin-3) and Disease Free Survival (DSF) (defined as the time from diagnosis to the first observation of disease progression or death from any type of case) in order to improve a more appropriate stratification of patients with urothelial carcinoma in advanced stage and a customization of treatment.

## Materials and Methods

### Ethic Statements

We used tissue samples from human participants. All tissue blocks have been previously declaired to be available for the purposes of the actual study by the Istitutional Review Board. This study was conducted according to the Declaration of Helsinki. The study was approved by the University Hospital of Verona Institutional Review Board (S1 File), approval number n.23/2013. Tissue blocks used in this study were collected as a part of routine care, and all donors provided written informed consent.

The full name of the ethics committee/Institutional Review Board: University Hospital of Verona Institutional Review Board.

The samples analyzed from the 74 patients exist before the study began and were collected for routine care by MB and GM.

All co-authors have access to any patient identifying information.

### Patients and samples

Patients previously resected for muscle-invasive or metastatic urothelial carcinoma at the Department of Pathology the University Hospital of Verona from 1995 to 2012, whose paraffin blocks were available for the study, were considered eligible. Only 40 of 46 patients (87%) were treated with chemotherapy: 34 patients (74%) with cisplatin 70 mg/m^2^ ev on day 2 plus gemcitabine 1000 mg/mq ev on days 1,8,15 every 28 days; 6 patients (13%) with carboplatin AUC 5 on day 1 plus gemcitabine on days 1,8 every 21 days due to a non optimal renal function; 6 patients (13%) were defined unfit for chemotherapy. A revision of the histological preparations with regard to the pathological grading and staging was accomplished. Formalin fixed-paraffin embedded blocks from bladder urothelial carcinomas were retrospectively retrieved from the archives and 3 tissue microarrays (TMA) were built.

### Tissue microarrays (TMA)

Each tumor was sampled 3 times and paired with normal urothelium. The whole slides were reviewed and appropiate tumour points were selected for each case in order to build representative tissue microarray paraffin master blocks. Three cores of neoplastic tissue and two cores of normal urothelium adjacent were available for each case of muscle invasive urothelial carcinoma. We performed three samples for each piece per lymph node metastases, in cases with lymph node metastasis, histologically diagnosed.

### Immunohistochemical analysis

**Fig 1:**
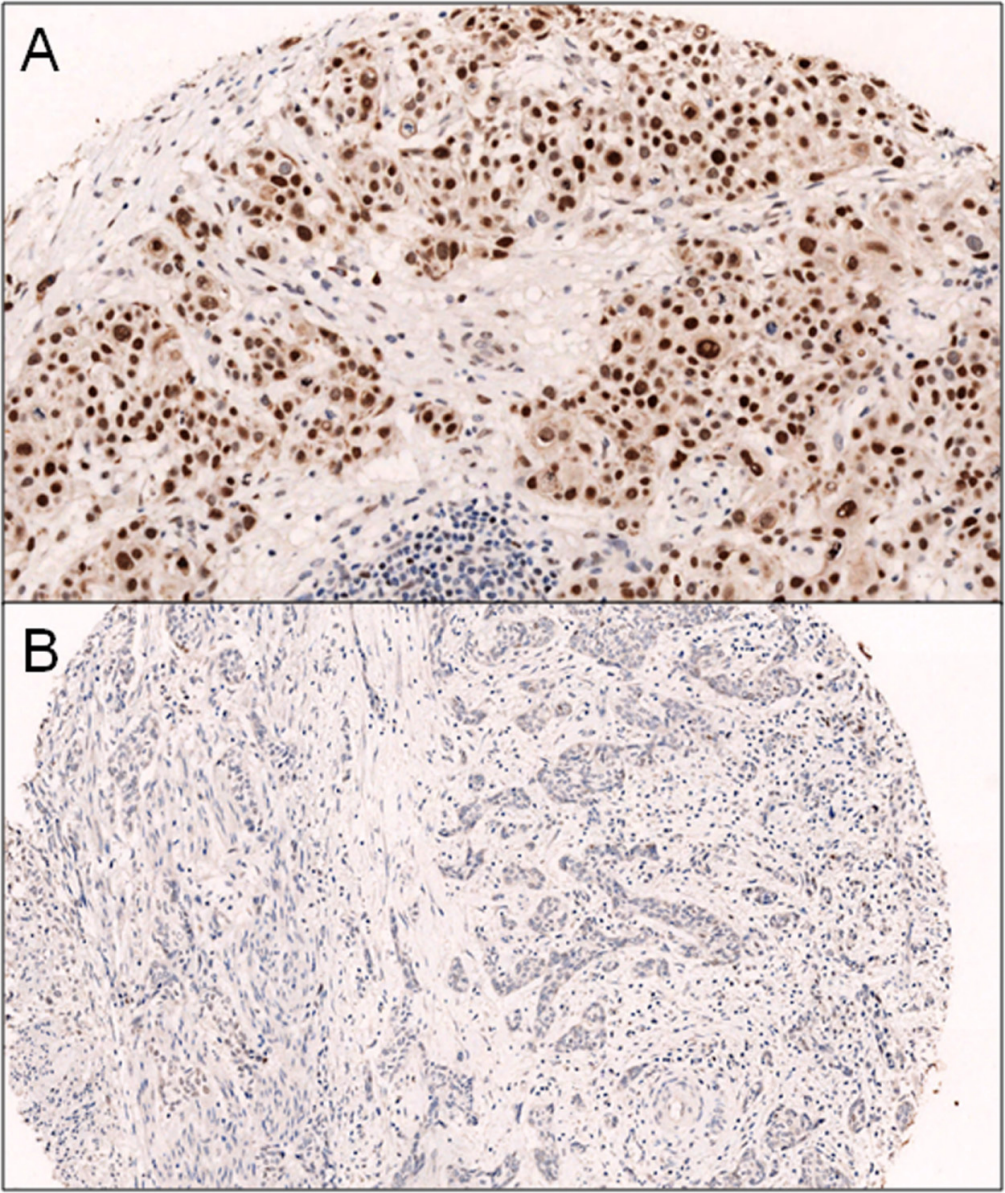
A] c-Myc iperexpression (2+); B] absence of expression of c-Myc.

**Fig 2:**
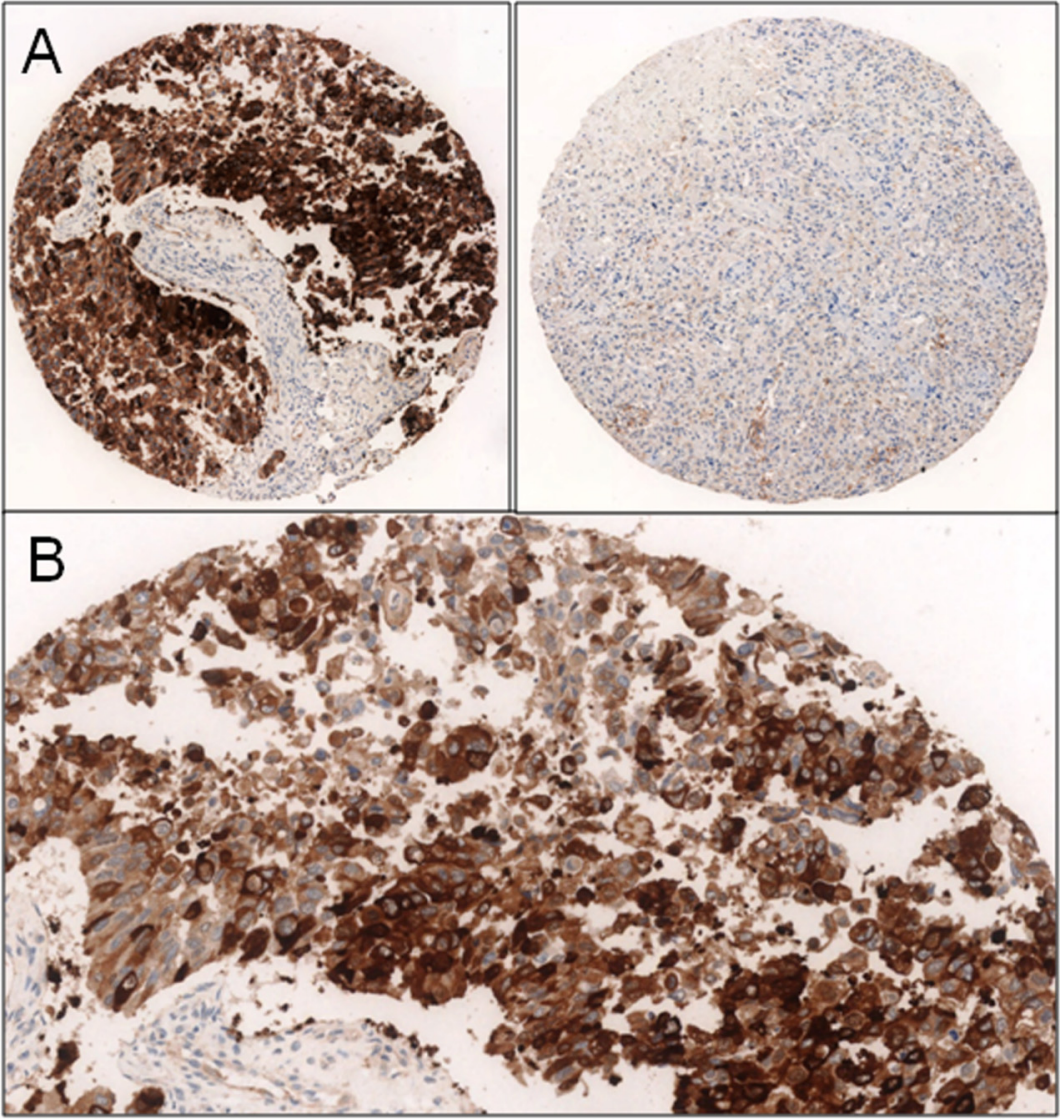
A] strong positive beta-3-tubulin expression versus absence cores B) 2+ beta-3-tubulin expression.

Immunohistochemical expression of c-Myc (Y69, 1:300, Epitomics, Burlingame) and beta-tubulin-3 (3F3, 1:40000, Seven Hills, Bioreagents, Cincinnati) was evaluated. All the sections were developed with the Envision peroxidase detection system (DAKO, Carpinteria, CA), a technique very sensitive and capable of preventing false positivity due to endogenous biotin present in the tissues. The c-Myc was considered positive when expressed at the nuclear level and classified into three groups: 0 (negative), 1 + (>15% weakly positive cells), 2 + (>15% strongly positive cells) (Fig 1). The beta-tubulin-3 was considered positive when expressed at the level membranous and cytoplasmic as follows: 0 (negative), 1 + (>15% weakly positive cells), 2 + (>15% strongly positive cells) (Fig 2). For beta-tubulin-3, we have also reported a positive score in the reactive tissue adjacent to the tumor. In order to simplify the results and choose a single representative value for each variable, we reported the median of the number of observations corresponding to each patient. The median score per case was used for the statistical analysis.

### Statistics

Descriptive statistics was used to summarize pertinent study information. Follow-up was analyzed and reported according to Shuster [7]. The correlation between variables were analyzed according to chi-square, Student's *t*, and Mann–Whitney (nonparametric) tests. The hazard ratio (HR) and the 95% confidence intervals (95% CI) were estimated for each variable using the Cox univariate model [8,9]. A multivariate Cox proportional hazard model was developed using stepwise regression (forward selection, enter/remove limits *P* = 0.10 and *P* = 0.15), to identify independent predictors of outcomes. The assessment of interactions between significant investigation variables was taken into account when developing the multivariate model. Event-free survival (EFS) and overall survival (OS) were calculated by the Kaplan–Meier product limit method from the date of the surgery until death due to cancer or death for any cause [10]. The log-rank test was used to assess differences between subgroups. Significance was defined at the *p*<0.05 level. The SPSS (18.0), and MedCalc (10.0.1) licenced statistical programs were used for all analyses.

## Results

### Clinico-pathological findings

The clinico-pathological data of the included 74 patients are summarized in Table 1. Median age was 67 years (range 44–86). Histological samples from 52 of the 74 patients was recovered in the context of 9 biopsies (17.3%) and 43 surgical specimens (82.7%) of radical cystectomies and available for subsequent analysis. Of the 52 series of slides reviewed, those belonging to 4 patients were found for the quality and quantity of material not sufficient to the study and therefore excluded from the subsequent analysis.

**Table 1:** Patients’ characteristics, according to clinicopathological and molecular variables.

Variables	Number	Rates (%)
Sex		
Male	52	70.3
Female	22	29.7
Comorbidity		
Yes	63	85.1
No	11	14.9
Staging		
T1-2	19	25.7
T3-4	51	68.9
Unknown	4	5.4
Lymph-nodal status		
Positive	31	41.9
Negative	19	25.7
Unknown	24	32.4
Grading		
G1-2	8	10.8
G3	42	56.8
Unknown	24	32.4
Vascular invasion		
Yes	34	45.9
No	40	54.1
Neural infiltration		
Yes	16	21.6
No	58	78.4
Type of surgery		
Cystectomy	55	74.3
TUR	18	24.3
Unknown	1	1.4
C-MYC expression (IHC)		
0	29	39.2
1	13	17.5
2	4	5.5
Unknown	28	37.8
Tubulin expression (IHC)		
0	14	18.9
1	15	20.2
2	17	37.8
Unknown	28	37.8

G: grade; TUR: trans-urethral resection; IHC: immunohistochemistry. Among 46 cancer, 36 showed urothelial, 2 squamous, 2 sarcomatoid, 2 micropapillary, 1 squamo-glandular, 1 small cell, 1 nested, 1 plasmocytoid differentiation.

### Tissue microarray (TMA) master blocks

Three mDigita il testo o l'indirizzo di un sito web oppure aster TMAs comprising 287 cores were obtained, 177 of which taken from tumor tissue, 76 from normal urothelium adjacent to the tumor and 34 controls. Of the 177 cores, 33 samples from lymph node metastases were punched from 11 of 48 patients.

### Immunophenotypical profiling

Overall, 46 patients for c-Myc and beta-tubulin-3 were available for final score as the microscopic evaluation of cores from 2 of the 48 patients, originally selected truly were representative of normal/dysplatic urothelial tissue (the other two cases were lost materials during tissue sectioning). Among 46 cancer, 36 showed usual urothelial, 2 squamous, 2 sarcomatoid, 2 micropapillary, 1 squamo-glandular, 1 small cell, 1 nested, 1 plasmocytoid differentiation. A total of 10 lymph nodes were finally available, as sampling by 1 of the 11 lymph nodes were composed by whole normal lymphoid tissue. The analysis of lymph node metastasis has detected the prevalence of negative cases (6/10 cases, 60%) compared with the positive (1/10 cases of weak positivity, 10%; 3/10 cases of strong positivity, 30%) with a discrepancy of expression of the marker in 4 of 10 cases (40%): 1 case is shifted from negative to positive, and 3 cases from weak positive (weak 1 and strong 2) to negative. c-Myc was positive in 17 cases out of 46 (37%), 13 of these showed a weak positivity (28%) and 4 a strong positivity (8%), while 29 of 46 cases were negative (63%). Lymph node metastases were strongly positive in 20% of cases (2/10), weakly positive in 20% (2/10) and negative in the remaining cases (6/10, 60%). In 5 patients it was possible to observe a discrepancy between the expression levels of c-Myc in the primary tumor and those in lymph nodal metastasis: in 2 cases the strong expression turned from negative to positive, in 2 cases from negative to weak positive and in 1 case from weak positive to negative. The expression of beta-tubulin-3 was strongly positive in 17 cases out of 46 (37%), weakly positive in 15 cases out of 46 (33%) and negative in 14 cases out of 46 (30%). The nodal positivity was instead found in 4 of 10 cases (40%), while the remaining are negative in 50% of cases (5/10) and weak in 10% (1/10). Even for the expression of this marker were found some discrepancies between the primary tumor and lymph node metastasis: in 2 cases the expression shifted from negative to positive, and in 1 case from positive to negative.

### Data analysis

**Fig 3:**
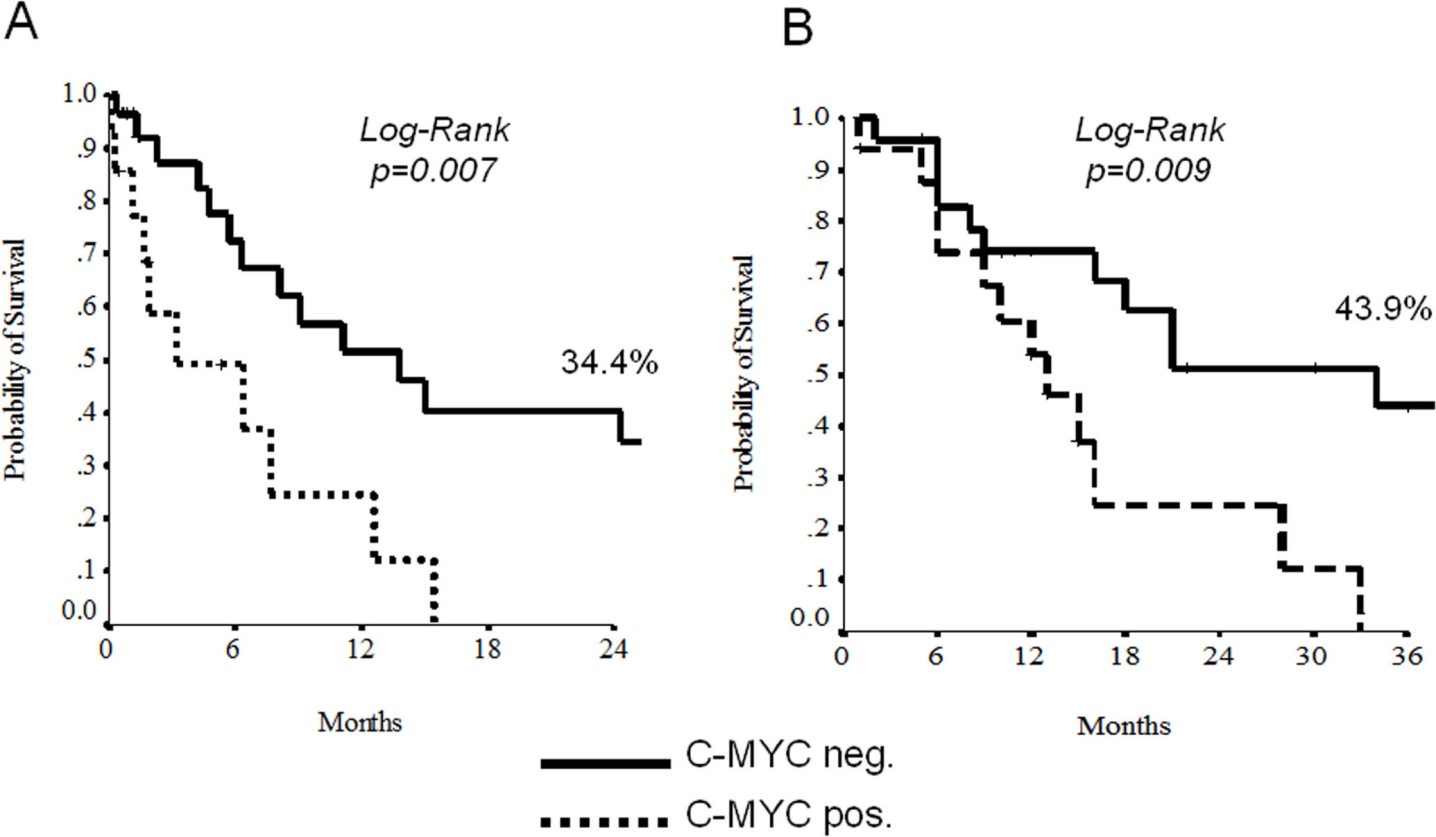
Unudjested Kaplan-Meier curves for Event-free Survival (A) and Overall Survival (B) according to c-Myc.

**Fig 4:**
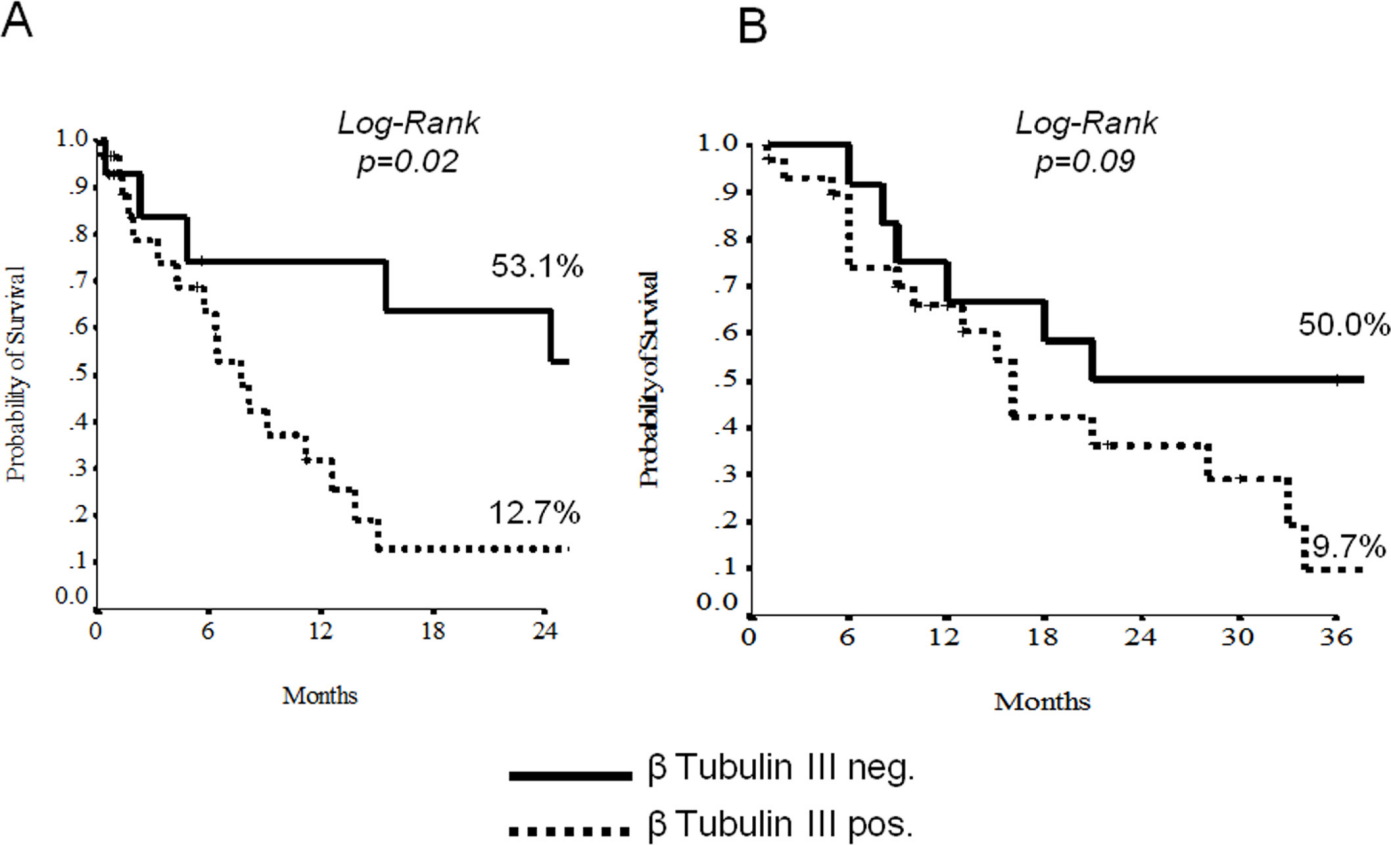
Unudjested Kaplan-Meier curves for Event-free Survival (A) and Overall Survival (B) according to beta-3-tubulin.

**Fig 5:**
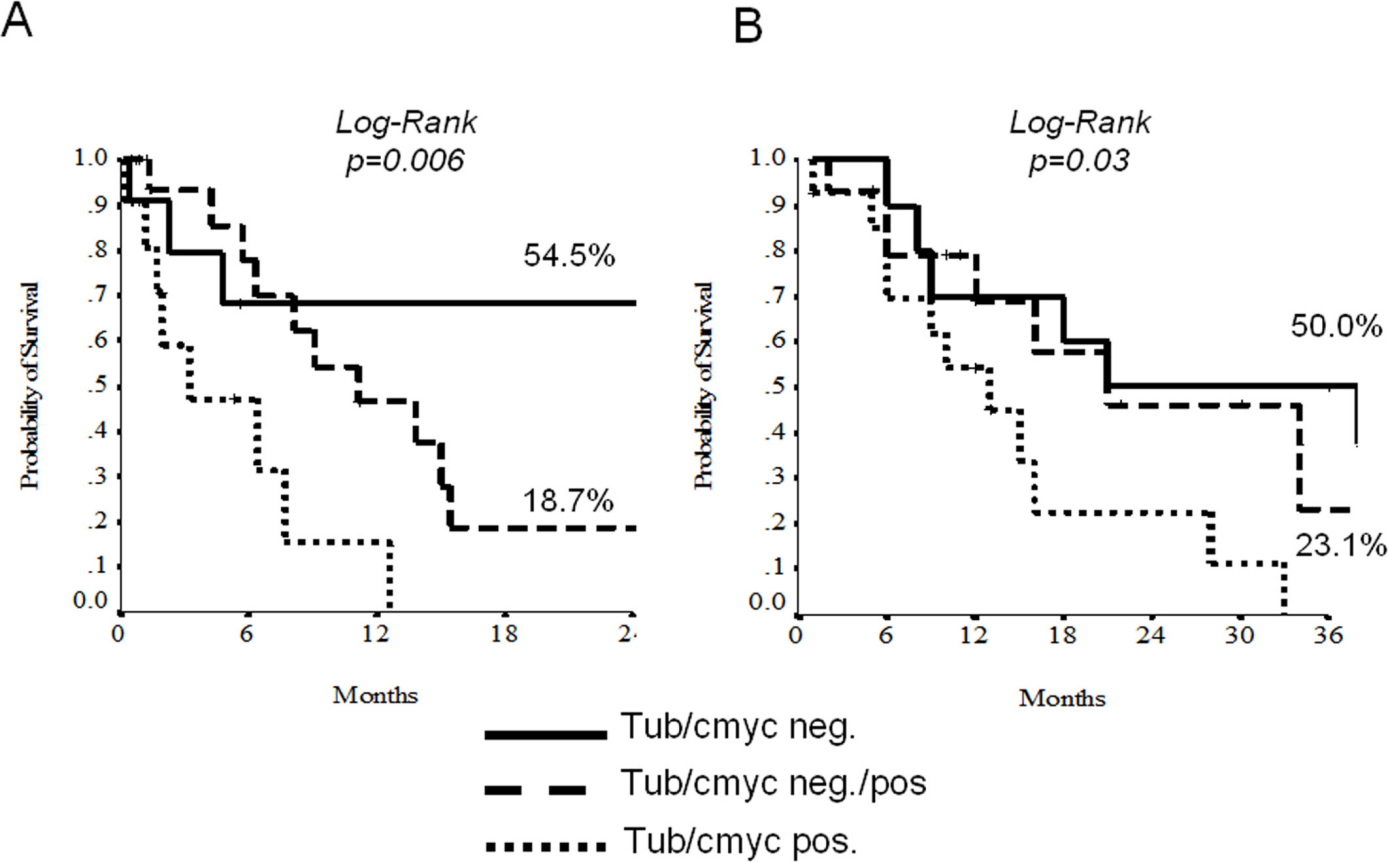
Unudjested Kaplan-Meier curves for Event-free Survival (A) and Overall Survival (B) according to the combination of c-Myc and beta-3-tubulin.

At the univariate analysis, node-involvement, c-Myc and beta-tubulin-3 overexpression demonstrated to discriminate patients for a shorter EFS and OS (Tables 2 and 3, Figs 3 and 4). The 11 patients displaying negative beta-tubulin and c-MYC had a statistically significant better 2-yrs EFS and OS than those with mixed expression (19 patients) or double positivity (11 patients) (Figs 4 and 5). Results with regard to T-size and nodal involvement are retorted in Table 4.

**Table 2:** Univariate analysis for Event Free Survival (EFS).

Factor	Hazard Ratio	95% CI	p-value
Sex	1.92	0.97–3.77	0.058
Age	1.23	0.67–1.26	0.48
Comorbidity (yes vs no)	2.16	0.67–6.07	0.14
Tumor Size (T3-4 vs T1-2)	1.23	0.62–2.44	0.53
Nodal involvement (positive vs negative)	2.19	1.02–4.69	0.043
Grading (G3 vs G1-2)	2.04	0.78–5.39	0.14
Vascular invasion	1.07	0.58–1.96	0.83
Neural infiltration	1.48	0.76–2.88	0.24
Adjuvant chemotherapy	1.92	0.68–5.40	0.21
c-MYC (IHC score 1+/2+ vs0)	3.05	1.29–7.21	0.011
Tubulin (IHC score 1+/2+ vs0)	3.10	1.16–8.25	0.024

vs: versus; G: grade; IHC: immunohistochemistry.

**Table 3:** Univariate analysis for Overall Survival (OS).

Factor	Hazard Ratio	95% CI	p-value
Sex	1.01	0.48–2.12	0.97
Age	2.47	1.19–5.12	0.015
Comorbidity (yes vs no)	1.12	0.46–2.71	0.80
Tumor Size (T3-4 vs T1-2)	1.48	0.66–3.31	0.33
Nodal involvement (positive vs negative)	3.11	1.04–9.31	0.042
Grading (G3 vs G1-2)	7.73	1.01–15.9	0.049
Vascular invasion	1.33	0.68–2.62	0.40
Neural infiltration	1.14	0.51–2.53	0.73
Adjuvant chemotherapy	1.65	0.50–5.41	0.40
c-MYC (IHC score 1+/2+ vs0)	2.94	1.23–7.01	0.015
Tubulin (IHC score 1+/2+ vs0)	2.06	0.89–4.96	0.10

vs: versus; G: grade; IHC: immunohistochemistry.

**Table 4:** Outcome results according to tumor size and nodal involvement.

	**Beta-tubulin**	**DFS (2-yrs)**	**p-value**	**OS (3-yrs)**	**p-value**
T 1–2	Negative	n.e.	n.e.	n.e.	n.e.
	Positive	n.e.		n.e.	
T 3–4	Negative	41.7	0.13	40.0	0.38
	Positive	15.7		12.6	
Node-negative	Negative	66.7	0.05	40.0	0.32
	Positive	12.5		0.0	
Node-positive	Negative	20.8	0.98	40.0	0.77
	Positive	20.6		14.0	
	**c-MYC**	**DFS (2-yrs)**	**p-value**	**OS (3-yrs)**	**p-value**
T 1–2	Negative	33.3	n.e.	n.e.	n.e.
	Positive	n.e.		n.e.	
T 3–4	Negative	34.5	0.07	34.5	0.07
	Positive	0.0		0.0	
Node-negative	Negative	33.8	0.72	50.0	n.e
	Positive	0.0		n.e.	
Node-positive	Negative	26.0	0.79	31.8	0.90
	Positive	0.0		0.0	

DFS: disease free survival; OS: overall survival; yrs: years; n.e.: not evaluable (too few patients).

Overall 5-yrs DFS and OS were 11.7% and 30.2%, respectively.

## Discussion

In our study we conclude that: 1) 37% of patients with urothelial carcinoma in advanced stage do express c-Myc; 2) c-Myc is a negative prognostic factor, in fact, its expression results in recurrent disease less than 2 years of diagnosis (p = 0.007; 3) the expression of beta-tubulin-3 turns out to be a negative prognostic factor for disease recurrence within 2 years from diagnosis (p = 0.02); 4) the molecular pathway of co-expression of c-Myc and beta-tubulin-3 stratify the prognosis of patients with urothelial carcinoma in advanced stage (p = 0.006); 5) c-Myc pathway deserves further investigation as predictive marker of response to targeted therapies.

The need to focus on urothelial carcinoma in advanced stage arises from the evidence that this tumor has a poor prognosis in a significant group of patients. The classic morphological parameters (such as histology, stage, grading, vascular invasion and neural infiltration) actually show a limit in predicting the survival of these patients. While for some neoplasms originating from other districts (such as the gastric tract, breast, lung and central nervous system) were identified molecular pathways useful for the stratification of patients and set of tests measuring protein or DNA abnormalities, such as Her-2, ALK, or 1p and 19q status [11–13]. Robust and reproducible biomarkers are not available for personalized management of the patient [3].

Some drugs that inhibit the pathway of c-Myc have shown promising results in other cancers but have been little studied in bladder urothelial carcinoma [14]. Similarly, spindle poisons drugs (such as taxanes), which inhibit mitosis by binding tubulin, have not been extensively investigated in urothelial tumors. For these reasons, we sought to test these markers on formalin-fixed tissue by immunohistochemical methods in order to reveal any protein expression, promising after correlation with clinical endpoints, for better prognostic stratification of patients.

Our data show that the stratification of positivity for c-Myc and beta-tubulin-3 is an important prognostic factor for DFS; we also propose the use of different expression of these markers as an indicator for the stratification of patients in clinical trials that take into consider the validation of the efficacy of drugs inhibiting these markers.

The proto-oncogene c-Myc encodes a transcription factor that allows the cell to start the cell cycle, through the activation of several genes involved in proliferation [15]. It is often overexpressed or mutated in many human cancers and it is frequently associated with aggressive tumor behavior and poor cell differentiation. The renewed interest in the Myc family as a possible therapeutic target, has been favored by recent genetic studies showing that a temporary systemic inhibition of this transcription factor has a significant therapeutic effect on different types of neoplasia [16]. The proteins of the Myc family in fact, contrary to many classic therapeutic targets, are not easily replaced by other signaling pathways, often a mechanism at the basis of a resistance to target therapy. Some studies have investigated the overexpression of c-Myc protein in urothelial carcinoma showing a significant association between the increase in the number of gene copies and tumors of advanced stage or high grade, while little is known about the possible prognostic value of c-Myc [17–19].

Our work highlights a positive expression of c-Myc in 37% of patients and a statistically significant correlation between its expression levels and 2-yr DFS, which results reduced in cases with positive expression. These results may provide the rationale to identify at molecular level patients with poorer prognosis eligible for clinical trials with c-Myc pathway inhibitors.

Tubulin is a globular protein which is the fundamental unit of the structures of the cytoskeleton called microtubules. It is present in the cytoplasm in the form of dimer α / β. In particular, the beta-tubulin-3 was proposed as a possible biomarker predictive of response to taxanes. In fact, some studies argue that overexpression of beta-tubulin-3 may be a common mechanism of resistance to many advanced solid tumors [20–22]. The predictive and prognostic role of beta-tubulin isotypes expression in urothelial bladder carcinoma has been recently evaluated, suggesting an interesting association between high beta-1-tubulin, beta-2-tubulin, beta-tubulin-3 expressions and unfavourable clinico-pathologic factors (like tumour grade and stage), and shorter recurrence-free survival [23].

The results of this study highlight the existence of a statistically significant correlation between the expression of beta-tubulin-3 and the 2-yr DFS, thus patients with primary tumors were negative for this protein in disease-free survival times greater than the positive counterpart. It has not been possible to evaluate the correlation between the expression levels of beta-tubulin-3 and the response to taxanes as in our cases only 5 patients were subjected to a treatment with these drugs and, of these, only one patient in the forefront while for others it was a second or even third line, often without the ability to complete therapy for the arrival of death.

Finally, another important aspect is that all aforementioned molecules may be also tested on tissue from biopsies.

The combination of different molecular pathways arise from the need to constantly improve prognostic stratification. Emerging diagnostic tools show that single biomarkers may not be sufficient to display the biological behaviors [24]. Easy techniques available in routine practice such as the immunohistochemical analysis permit the combination of double staining such as those nuclear (i.e. c-Myc) and cytoplasmic (i.e. beta-tubulin-3) markers per single reaction. Being beta-tubulin-3 a prognostic biomarker and the c-Myc pathway a potential innovative tool for new generation targeted therapy, we highlight from our study these two molecules be promising for additional translational research.

## Supporting Information

S1 File Ethics committee approval. (DOC)
