# Supplementary material for: Prognostic Value of Beta-Tubulin-3 and c-Myc in Muscle Invasive Urothelial Carcinoma of the Bladder
Source: PLoS One. 2015 Jun 5;10(6):e0127908. doi: 10.1371/journal.pone.0127908 (PMC4457798; doi:10.1371/journal.pone.0127908)
Supplement: S1 File — (DOC) [file pone.0127908.s001.doc]

S1 File. Ethics committee approval

Study name: **Prognostic value of beta-tubulin-3 and c-Myc in muscle invasive urothelial carcinoma of the bladder.**

The study “**Prognostic value of beta-tubulin-3 and c-Myc in muscle invasive urothelial carcinoma of the bladder”** is approved with code n.23/2013 by the Institutional Review Board. The full name of the ethics committee/Institutional Review Board - University Hospital of Verona Institutional Review Board: Prof. Guido Martignoni and Prof. Matteo Brunelli.

We hereby declare that there are no ethical or legal restriction on our data.

I confirm whether the data are available from the paper, ALONE.
